# Consensus on curriculum design for a person-centred leadership learning programme—a delphi study

**DOI:** 10.3389/frhs.2026.1866482

**Published:** 2026-06-30

**Authors:** Deborah Jane Baldie, Shaun Cardiff, Kate Sanders, Giselle Cope, Bibi Hølge-Hazelton, Mette Kjerholt, Tanya McCance

**Affiliations:** 1 NHS Grampian, Aberdeen, United Kingdom; 2 Robert Gordon University, Aberdeen, United Kingdom; 3 Fontys Hogescholen, Eindhoven, Netherlands; 4 Foundation of Nursing Studies, London, United Kingdom; 5 Research Support Unit, Zealand University Hospital, Koege, Denmark; 6 Department of Regional Health Research, University of Southern Denmark, Odense, Denmark; 7 Department of Hematology, Zealand University Hospital, Roskilde, Denmark; 8 Ulster University, Coleraine, United Kingdom

**Keywords:** curriculum, education, expert consensus, leadership, person-centred

## Abstract

**Introduction:**

The creation of person-centred cultures in health care settings is understood as a pre requisite to the provision of person-centred practice. Such cultures need systematic facilitation by health care leaders. While there are few empirically informed theoretical models for person-centred leadership and many organisations internationally, educate leaders in how to transform cultures to become more person-centred, there is lack of consensus on how person-centred leadership can be developed and no high quality, multi-centre evaluations of their effectiveness or impact. Before such evaluations can be undertaken an agreed curriculum is necessary. Only by having this can the fidelity of a leadership programme, subject to evaluation, be maintained.

**Methods:**

This study sought to gain international expert consensus on a curriculum for person-centred leadership using a modified Delphi method. Delphi studies employ anonymous and confidential survey methods to ensure expert consensus can be achieved without the interference of influential voices or group coalitions. They typically include two or more rounds with controlled feedback between rounds. We developed curriculum components by undertaking a literature review to identify programmes and theoretical models for person-centred leadership alongside a mapping of current person-centred leadership programmes being delivered by members of an international community of practice committed to the development of person-centred practice. We recruited experts using snowball recruitment. In round 1 we focused on seeking consensus on 16 curriculum components and 6 pedagogical delivery principles and offered the opportunity to comment on the phrasing or clarity of any components or propose additional components.

**Results:**

Round 1 resulted in consensus of ≥70% for all components. All bar one had consensus of ≥80%. In round 2 consensus was achieved for all components (≥80% and a mode of 5).

**Discussion:**

Using a modified Delphi study approach we have developed an internationally critiqued curriculum framework for person- centred leadership programmes, specific enough to ensure the fidelity of future programme delivery whilst providing sufficient flexibility for appropriate cultural modification within any complex intervention evaluation study. Research is now needed to evaluate the impact of engagement with this curriculum on healthcare leaders’ self-views; enacted leadership behaviours and practices; health care cultures and other relevant patient and staff outcomes.

## 1 Introduction

Creating person-centred cultures within health care teams is seen as a prerequisite to consistently delivering person-centred practice (1). Patient-centred practice, defined as “providing care that is compassionate, empathetic, and responsive to the needs, values, and expressed preferences of each individual patient [as an informed decision maker in their care]” (2) is directly linked to better patient satisfaction, well-being and self-management, with meaningful care relationships achieved through professionals demonstrating and authentic caring and respectful approach to patients as unique individuals (2, 3). Person-centredness is conceptually similar to patient-centred care but becomes more inclusive when the complexity of healthcare is taken into account through a systemic and relational lens (4). McCance & McCormack (5) defined person-centred practice as “an approach to practice established through the formation and fostering of healthful relationships between all care providers, service users and others significant to them in their lives. It is underpinned by values of respect for persons (personhood), individual right to self-determination, mutual respect and understanding. It is enabled by cultures of empowerment that foster continuous approaches to practice development”. However, the concept of person-centred care/practice has not been fully adopted into (inter)national discourses, which may explain the limited empirical evidence of the positive outcomes. For instance, in Europe the concept is most commonly found in publications arising from countries using a Beveridge (tax-funded) model of healthcare (4). The development of person-centred practice is reported as being complex with barriers to be overcome including: transforming traditional practices and structures, stereotypical attitudes among healthcare staff and tailoring interventions for the particularities of the context and target groups (6, 7). Whilst many hospital, community and social care organisations and (research) projects start by focusing on person-centred care and the lived experiences of patients, clients and residents, McCance & McCormack (5) recommend equally focusing on the personhood and wellbeing of staff. Without person-centred cultures it is very difficult for health care practitioners to engage in person-centred ways of being, and thus for patients and their families to experience person-centred care (7). Person-centredness is a core value of workplaces that foster healthcare staff retention (8) and their front-line leaders are able to encourage staff intent to stay when they focus on developing relational connectedness (feeling safe, respected and belonging) and a healthful workplace culture (energy-giving for the benefit of health and wellbeing) (9). Person-centredness should also permeate the whole system as Thomas et al. (10) found that “caregiver perspectives and behaviour are unlikely to change if the organisation as a whole does not embrace a person-centred approach towards the caregivers”.

Person-centred cultures do not merely emerge, they require sustained and systematic facilitation from healthcare leaders who draw on theoretically and empirically informed knowledge (1, 11–13). It is logical to assume that health and social care leaders aiming to foster person-centred cultures and care, should also integrate the essence of person-centredness into their leadership practice. Lynch (14) states that situational, clinical leaders who have embodied the essence of person-centredness diagnose staff performance, competence and commitment to person-centred care before engaging in leadership behaviour to foster person-centred care. Consequently, leaders can be flexible in their partnering facilitation: inclusive and non-confrontational as they enable each individual practitioner to progressively move through multiple developmental stages in (co-)creating person-centred environments and care with their colleagues: from being hesitant and feeling incompetent, to being willing and able. Amidst contemporary cultures of “quick fixes” in health and social care settings where problem-solving interventions are quick and seldom address deeper underlying issues, McCormack (15) argues that there is urgent need for person-centred leadership development that enables systematic and sustainable action for person-centred cultures and care.

For several years, numerous members of the Person-centred Practice International Community of Practice CIC (PCP-ICoP) have individually been involved in creating and delivering practice development and leadership programmes rooted in transformational practice development i.e., “fundamentally about person-centred practice that promotes safe and effective workplace culture where all can flourish” (16). While there is broad consensus among the PCP-ICoP and small, qualitative service evaluations demonstrating a positive impact of such programmes on practitioners and practice, there has not been any systematic, large-scale programme evaluation. This position reflects the state of evaluation of other leadership programmes delivered within health and social care settings. A recent review of the state of leadership programme evaluation in the NHS in England, for example, has highlighted that while it is estimated that millions of pounds are spent providing leadership training, there is yet to be strong empirical evidence of their impact in terms of measurable improvements in care delivery (17). Lood et al. (18) have analysed curriculum leads’ and facilitators’ reflections on the development and delivery of a person-centred leadership programme, but did not publish details of the curriculum content, its theoretical origins or the development process. To conduct any (large scale) complex intervention evaluations such as a person-centred leadership programme (PCLP), a clear definition of the intervention needs to be in place (19).

Whilst several authors (14, 20, 21) have provided detailed descriptions and conceptual frameworks for person-centred leadership, no consensus process has been published as to what content should be included in an educational curriculum designed to develop person-centred leaders. Defining such a curriculum is fundamental to ensuring fidelity of programme delivery for future complex intervention evaluation studies. The Delphi study reported in this paper aims to address this issue by reporting the outcomes of a consensus process, among international experts on the content for a person-centred leadership curriculum framework. The study is reported here in accordance with the Equator guidelines RG_DELPHISTAR_engl-1.pdf.

## 2 Methods and materials

### 2.1 Aim

The aim of this paper was to seek consensus for the components parts of a Person-Centred Leadership Curriculum Framework (PCLCF) from an international panel of experts in person-centred practice and leadership development.

### 2.2 Study design

The Delphi technique is a method for generating, refining and synthesising expert opinions in a systematic manner using multiple structured rounds, and so addresses incomplete knowledge and uncertainty on complex issues (22, 23). It was chosen to support a research team in moving current “talk” about numerous bespoke, regional or country specific PCLP curricula (i.e., partial knowledge) to an internationally agreed curriculum that could then be used for the development, delivery and future evaluation of person-centred leadership development programmes.

Modified Delphi studies draw on principles of constructivist epistemology. Whilst acknowledging that there is no absolute “true” knowledge, consensus is sought on what “is” thereby strengthening and expanding situated knowledge through a process of co-production (23). It is a recognised research method offering equity of voice through anonymity and minimising the influence of dominant voices or coalitions within an expert group (23). Another key feature of the modified Delphi technique is the adjustment of round one from open questions used in a traditional Delphi to inclusion of predefined questions determined from previous literature or other sources of knowledge. The existence of a range of current curricula; a person-centred leadership model and previous research determining the underlying principles of person-centred curricula (24) meant that a modified technique was more aligned to the contextual starting point for this research and afforded the ability to co design with a range of international experts in the field of person-centred practice and leadership, keeping all voices equal.

The study protocol was peer reviewed by an academic in nursing education who was not a member of the research team, and further reviewed by the institution's ethics committee. Participant quasi-anonymity was safeguarded by coding participants and restricting access to participant details to the primary investigator. To minimise potential coercion, participation was voluntary with no recruitment approaches being made to potential participants directly by the research team. Feeding back the findings to participants per round, helped ensure reliability of interpretations.

### 2.3 Development of a tentative curriculum

The starting point for this Delphi study was the development of a tentative PCLCF consisting of curriculum components and pedagogical principles. Seven PCP-ICoP members from four European countries, working across healthcare organisations, higher education and third sectors and a keen interest in evaluating PCLPs as well as experience of delivering person-centred leadership programmes formed the research team (the authors). Together they co-constructed the tentative PCLCF components i.e., the round 1 questions.

The process of identifying curriculum components included a review of existing literature on person-centred leadership research and theory (1, 12, 13, 21) and existing person-centred leadership programmes being facilitated by the research team in Scotland, England, Northern Ireland, The Netherlands, and Denmark.

The literature review focused on empirical studies published in the last 10 years which related to the creation of, delivery and/or evaluation of leadership programmes designed to support the development of person-centred leaders OR person-centred leadership theories, models or frameworks OR papers relating to examination of person-centred leaderhsip in action. A search on google scholar for “person-centred leadership” and not “leading person-centred care”, between 2014 and 2024, revealed very few hits (14, 21, 36, 37). Only papers which included details of person-centred theories, person-centred leadership in action or person-centred leadership programmes were included. Literature known by members of the research team that specially refers to person-centred leadership, was also included in the review process (12, 13). All members of the team read at least 3 publications and several meetings ensued to a) share the content of their curricula, and b) share their reading of the essential factors of person-centred leadership. Findings were further analysed by two co-investigators, who themed the key factors associated with person-centred leadership and member-checked the framework with the rest of the research team. The four themes were: embodying person-centredness and leadership as relational phenomena; developing the leading of self; developing the leading of others and developing the leading of self and others in dynamic contexts. In pairs, the research team rephrased and refined the component parts into curriculum statements and team consensus sought during a subsequent workshop. When all agreed that the components accurately reflected issues core to person-centred leadership and the language used enabled ease of understanding by all, the framework was further augmented with philosophical and pedagogical principles from the person-centred curriculum framework (24) The tentative PCLCF was now ready for further refinement through the Delphi process.

We were only able to identify one published theory of person-centred leadership. It was developed through action research in health care for nursing leaders (21). Many of the research team had used it in their leadership programmes and found it to be applicable across a range of health care professionals. This, combined with the fact that it was the only empirically developed framework, led the team to agree to it being used as the base theoretical framework to build the curriculum content.

Once initial charting was complete the team worked together in a workshop format to synthesise the data—reading the data extraction tables, looking for patterns and then grouping constructs into 4 themes: embodying person-centredness and leadership as relational phenomena; developing the leading of self; developing the leading of others and developing the leading of self and others in dynamic contexts. Researchers worked in pairs to transform each factor into a curriculum statement (components) and these were refined through a subsequent workshop until consensus was built—where all agreed that the components accurately reflected issues core to person-centred leadership and used language that enabled ease of understanding by all. The curriculum components were then augmented with philosophical and pedagogical principles for the person-centred curriculum framework (24).

### 2.4 The delphi process

The Delphi process consisted of repeated circulation of the PCLCF statements among experts, asking them to rate their level of agreement with each statement (25). Within each round, statements with less than 70% agreement, or with commentary considered valid by the research team, would be removed or reformulated before the next round of consensus seeking. Between round 1 and 2 participants received controlled feedback on the degree of consensus for each components, which they could then take into consideration when scoring in the following round. This enabling of increasing convergence of opinion typically results in two to three survey rounds before sufficient consensus is reached on all components (25).

**Figure 1:**
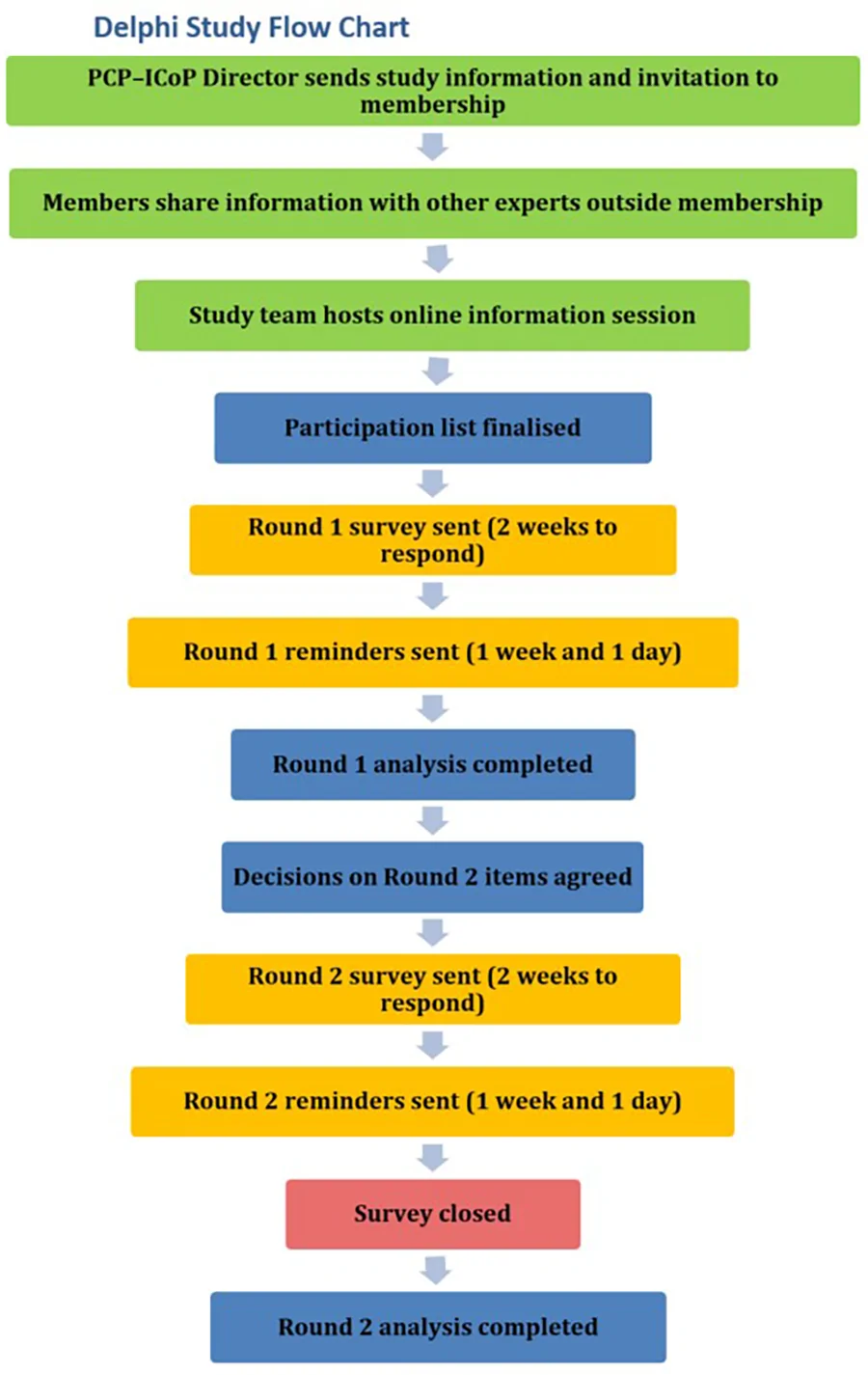
Delphi study process.

An illustration of the Delphi study structure can be found in Figure 1.

#### 2.4.1 Participant experts

A panel of experts in the field of person-centred practice (research, practice, leadership and education) was sought within the PCP-ICoP. Members were provided with an invitation email with the participant information leaflet by the Director of the PCP-ICoP, and invited to consider their participation using the following self-selection criteria:

- Facilitates person-centred leadership development for health care practice AND
- Demonstrates familiarity and use of the PCPF and its underpinning philosophies in their leadership work AND
- Demonstrates knowledge of a wide range of leadership theories AND
- Engages as health care leaders or educators with person-centred leadership development activities AND
- Demonstrates person-centred leadership in their practice

Members were also invited to share the study information with colleagues outside the PCP-ICoP in an attempt to widen the range of experts (26). Members of the research team were excluded from participating.

An on-line information session was then held to present the study and offer space to ask the research team questions. This enabled interested participants to make an informed decision to participate. If still interested, potential participants were asked to contact the principal investigator indicating their desire to participate in the study. All participants invited to participate in round 1 were also invited to participate in round 2. We did not collect data relating to experts’ roles or responsibilities to preserve’ anonymity within the study.

#### 2.4.2 Delphi rounds for data collection

In total, two Delphi rounds were undertaken between 25th June, 2025 and 10th October, 2025. In round 1 the questionnaire contained all 22 statements of the tentative PCLCF. After data analysis, participants received controlled feedback which consisted of whole group percentage consensus and mode per component, plus a high level overview of the changes made in light of open contributions (see Table 1). In round 2 participants were again invited to score their level of agreement with the refined and additional statements and subsequently received the findings from round 2 (see Table 2).

**Table 1:** Round 1 data.

Components no	Curriculum components	Percentage agreement	Mode
Embodying person-centredness and leadership as relational phenomena through:			
1	Exploring personal definitions of person-centredness and those defined in literature.	92	5
2	Exploring personal definitions of leadership and those defined in literature.	84	5
3	Melding and blending together definitions of person-centredness and leadership into a description of relational leadership practice.	84	5
Developing the leading of self through:			
4	Knowing self—exploring, transforming and/or embodying values, beliefs and assumptions associated with leading self and others within the workplace	72	5
5	Self-nurturing—enabling own coming into own/flourishing/muchness through self-compassion etc.	92	5
6	Resilience—growing one's ability to cope; openness and positivity; bravery, vulnerability and adaptability	84	5
7	Reflexivity—reflecting before-, in- and on-action, including one's own role within the situation	80	5
Developing the leading of others through:			
8	Relational processes for achieving a sense of connectedness—sensing (using one's senses to gather information about the current state of being, and verifying interpretations); balancing (the needs of other and self); contextualising (understanding the other as a person embedded in multiple roles and contexts); presencing (being there for the other person, and thinking with them); communing (engaging in an action-oriented dialogue).	88	5
9	Stancing—constantly and consciously re-positioning self in relation to other.	96	4
10	Exercising influence—horizontally and vertically within the organisational hierarchy.	84	4
11	Focusing on shared flourishing—leading (each) other towards mutual benefits such as: experiencing muchness and confidence; coming into own and job satisfaction; feeling valued, heard and supported; personal and professional (competency) growth, and trust.	88	5
12	Focusing on purpose—co-creating and leading towards a shared purpose.	96	5
Developing the leading of self and others in dynamic contexts through:			
13	Working with evaluations of practice (all quality dimensions: clinical, financial, staff etc).	92	5
14	Working sensitively with and within organisational and workplace cultures (understanding and morally responding to the influence of culture on one's leadership).	84	5
15	Purposefully (co)creating and (co)facilitating safe, critical and creative communicative spaces such as team building exercises; team meetings etc.	92	5
16	Promoting and fostering change for good i.e., Change which care staff and service users consider important and will enhance healthful cultures.	88	5
Pedagogical principles			
17	Working with adult/active, transformative and PC learning theory.	88	5
18	Offering choice of activities to meet the intended outcomes, thereby co-constructing the curriculum.	88	5
19	Using multiple sources of knowledge and pedagogical methods to develop learner leadership practice.	84	5
20	Using person-centred facilitation, based on relationality and reciprocity.	88	5
21	Encouraging high challenge and high support on each other's thinking, doing and becoming a person-centred leader.	84	5
22	Addressing the real world of practice by drawing on and applying learning in practice.	88	5

**Table 2:** Round 2 data—final PCLCF.

Components no	Curriculum components	Percentage agreement	Mode
Embodying person-centredness and leadership as relational phenomena through:			
1	Exploring personal definitions of person-centredness personhood and person-centred practice alongside those defined in literature.	100	5
2	Exploring personal definitions of professional leadership and those defined in literature	95	5
3	Melding and blending together definitions of person-centredness and leadership into a description of relational leadership practice.	86	5
Developing the leading of self through:			
4	Knowing self—exploring, transforming and/or embodying values, beliefs and assumptions associated with leading self and others within the workplace.	100	5
5	Enabling own well-being (coming into own/ muchness/ flourishing) through self-care, reflection and compassion	95	5
6	Growing individual ability to cope through openness and positivity, bravery, vulnerability and adaptability.	95	5
7	Reflexivity—reflecting before-, in- and on-action, including one's own role within the situation	100	5
Developing the leading of others through:			
8	Authentic and caring engagement with others to achieve a sense of relational connectedness—sensing (using one's senses to gather information about the current state of being, and verifying interpretations); balancing (the needs of other and self); contextualising (understanding the other as a person embedded in multiple roles and contexts); presencing (being there for the other person, and thinking with them); communing (engaging in an action-oriented dialogue).	95	5
9	Stancing—constantly and consciously re-positioning self in relation to other.	**81**	5
10	Exercising influence—horizontally and vertically within the organisational hierarchy.	90	5
11	Focusing on shared flourishing—leading (each) other towards mutual benefits such as: enhancing confidence, trust, job satisfaction and well-being (muchness and coming into own); feeling valued, heard and supported; growing personally and professionally (competency)	100	5
12	Focusing on purpose and vision—co-creating and leading towards a shared vision and purpose.	95	5
13	Facilitating the ongoing development of healthful cultures (workplace and organisational cultures conducive to the health and wellbeing of all)	95	5
Developing the leading of self and others in dynamic contexts through:			
14	Applying participatory and inclusive ways of evaluating and learning from practice (including professional practice, leadership practice and all quality dimensions: clinical, financial, staff etc).	95	5
15	Working sensitively with and within organisational, professional and workplace cultures (understanding and morally responding to the influence of culture on one's leadership)	95	5
16	Purposefully (co)creating and (co)facilitating safe, critical and creative communicative spaces such as team building exercises; team meetings etc.	95	5
17	Promoting and facilitating change for good i.e., change based on collective understanding and shared purpose which care staff and service users consider important and will enhance healthful cultures	90	5
18	Examining the criticality of the leadership i.e., working with power dynamics and an emancipatory intent	86	5
Pedagogical principles			
19	Working with adult/active, transformative and PC learning theory	100	5
20	Offering choice of activities to meet the intended outcomes, thereby co-constructing the curriculum.	86	5
21	Using multiple sources of knowledge and pedagogical methods to develop learners’ leadership practice.	100	5
22	Using person-centred facilitation, based on relationality and reciprocity	95	5
23	Encouraging high challenge and high support on each other's thinking, doing and becoming a person-centred leader.	100	5
24	Addressing the real world of practice by drawing on and applying learning in practice.	100	5

Headings in bold are curriculum domains.

For each round participants were sent an electronic link for the survey. Each component required a forced response using a five point Likert scale (1 = strongly disagree, 5 = strongly agree). Responding to all components was mandatory to complete the survey. Participants had two weeks to respond, and automated reminder emails were sent one week, and 1 day, before the closing date.

Round 1 had three aims: 1) building consensus on components which were deemed important to include in the curriculum; 2) adjusting the phrasing of components to enhance clarity of meaning; 3) identifying any additional components the research team had not included. Round 2 focused on gaining consensus on all components, with no facility for suggesting additional components or providing qualitative comment on those components which had received high consensus, or no commentary warranting adjustments to the phrasing of the components, in round 1.

### 2.5 Data analysis

In both rounds, frequencies were calculated for each answer category per component. The answer categories “agree and strongly agree” were pooled in order to calculate the percentage consensus for each component. Consensus was defined as 70% or more participants agreed that the component should be included in the curriculum. The mode for each component was also calculated in an attempt to assign ranking of each component, however, as the mode was consistently high, no ranking or sub group analysis was performed.

All qualitative comments offered in round 1 were categorised into confirmatory/non confirmatory feedback. Non confirmatory comments i.e., those which challenged the component or suggested a required change, were reviewed by the research team to determine need to alter the component and to agree any rephrasing.

## 3 Results

Twenty five participants were invited to participate in rounds 1 and 2 of the survey. All 25 responded in round 1 (response rate 100%) and 21 responded in round 2 (response rate 84%). Participants worked in a range of countries including UK and Northern Ireland; Republic of Ireland; Switzerland; Denmark; Germany; Sweden and Australia. Consensus across all components was achieved in round 2, therefore no further rounds were required.

### 3.1 Round 1 results

In round 1 participants were invited to rate their level of agreement with each curriculum component and pedagogical principle; provide comments on the phrasing or clarity of any item and suggest any additional components the research team had not included. Round 1 results can be found in Table 1. In this round there was agreement ≥70% for all components so none were removed. Only one component had consensus <80%. The mode for 16 components was 5 and the remaining 6 had a mode of 4.

We received 131 qualitative comments in Round 1, the majority (112) of which were confirmatory i.e., indicating strong agreement for the inclusion of the components. Examples of confirmatory comments:

I would prefer ‘facilitating’ instead of ‘fostering’, because I see facilitation central for leadership of person-centredness

WOW, I love this already. I endorse each of the options and strongly agree. I am super excited with what is to come. The statements are clear, succinct, relatable in language that resonates with me as a leader, clinician and educator

Due to the small number of non-confirmatory contributions, these were individually considered and discussed within the research team before any changes were made. Examples of non-confirmatory comments:

Is it necessary to specify ‘practice'? leadership practice? person-centred practice (patient work),.? What do you think about emphasising the Co-Creation?

Focusing on co-creation—leading towards a co-created and shared purpose. My thoughts: purpose orientation is included in almost all leadership theories. Co-Creation is not….

The research team, on reviewing round 1 data identified that 10 curriculum components and one pedagogical principle required refinement to aid understanding/ refine the comprehensiveness of each components. For example: component 1 in round 1 stated “exploring personal definitions of person-centredness”. Replies from participants to this component highlighted the lack of attention to the concept of personhood and its place as being fundamental to person-centred practice and leadership and the need to go beyond personal definitions/ conceptualisations. The component was refined to include this: “exploring personal definitions of person-centredness, personhood and person-centred practice alongside those defined in literature”.

Two new components were also formulated for Round 2. The first was the inclusion of “focusing on developing and sustaining healthful cultures (workplace and organisational cultures conducive to the health and wellbeing of all) as participants noted this as a key outcome of person-centred” practice and as such should be a focus of leaders seeking to create person-centred cultures of care. The second additional component was “examining the criticality of the leadership i.e., working with power dynamics and an emancipatory intent”. This was added in response to comments relating to the focus on emancipatory intent of this type of leadership; the need for the leader to critique their leadership style, decisions and actions to remain effective and authentic, examining themselves and their impact through a critical theory lens.

### 3.2 Round 2 results

In round 2, participants were asked to rate their level of agreement with 18 curriculum components and 6 pedagogical principles. Agreement ≥80% was achieved for all components, only falling below 90% for three curriculum components (components 3, 9 and 18) and one pedagogical principle (components 20). The mode for all components was 5 and therefore no components were removed nor ranked. Table 2 contains details of the data from round 2.

### 3.3 The curriculum framework

The final PCLCF detailed in Table 2 consists of 18 curriculum components and 6 pedagogical principles. Each of the curriculum components are situated within one of four overarching themes.

#### 3.3.1 Embodying person-centredness and leadership as a relational phenomenon

This first theme requires the learner to find meaning through considering personal and published definitions on person-centredness, personhood, professional and person-centred leadership and recognise it as a way of being rather than a set of tasks, skills and responsibilities.

#### 3.3.2 Developing the leading of self

This second theme has a deeper internal focus as the learner reflexively explores leading of self by recognising personal values, beliefs and assumptions. The source of reflections are scenarios of building relational connectedness with others, as well as caring for one's own wellbeing and ability to cope with the complexities of everyday practice. This reflexive learning starts to narrow the gap between actual leadership behaviours and desired person-centred leadership behaviours.

#### 3.3.3 Developing the leading of others

This third theme has a more external focus as the learner contemplates leading others and explores how continuously repositioning themselves whilst exerting influence can facilitate mutual flourishing, shared visions and a healthful workplace culture.

#### 3.3.4 Developing the leading of self and others in dynamic contexts

The last theme takes cognisance of the dynamic and challenging environments healthcare leaders work in, and they are facilitated in considering how they can support self and others to stay vlues based, critical, creative, responsive and purposeful in sustaining or changing the care environment for the good of all.

## 4 Discussion

We first provide an overview and discussion of the PCLFC and position it's purpose and value within current health and social care. This is followed by a critique of the Delphi process and we conclude by situating the curriculum framework within contemporary person-centred and relational leadership literature and theory, programme delivery considerations and the potential impact of future person-centred leaders. In particular it acknowledges the importance of relational connectedness and reflexivity to leaders developing healthful cultures in often challenging and complex health and social care contexts.

### 4.1 Overview and discussion of PCLFC

Paying attention to all four themes is important if we are to enable leaders to carefully consider how different theories of leadership can be enacted in the messy worlds of practice where context is often less than ideal. By positioning leadership as relational and leaders as always in a state of “coming into their own” (21)—in a state of being and becoming, leaders who engage with this curriculum have the potential to engage in changed leadership behaviours and ways of being in and sustain these changes while working in rapidly changing and demanding contexts.

Two core principles traversing all four themes are: continuous self-awareness and continuous development. Being and becoming a person-centred leader has no beginning nor end. As responsive and embedded persons, leader values and assumptions (and consequently behaviour) are more likely than not to evolve across time. Therefore the skills and methods acquired whilst engaging in a PCLP based on adult and transformative learning theory, should equip leaders in remaining self-aware and self-determinant as their leadership continues to evolve. When leaders experience the benefits of a supportive, critical yet non-judgmental learning environment based on person-centred learning theory within a PCLP, they should start to realise the inherent capacity each person has to direct their continued leadership development, and appreciate the mutual benefits of learning with and from others. Additionally, observing how person-centred facilitators on such programmes supports their individual and group learning could also offer learners insight into the skills and methods that they could adopt into their leadership whilst aiming to facilitate the development of person-centredness within the care environments they are leading.

The PCLCF components are consciously formulated to accommodate the pragmatism, co-constructionism, transformative and relationism principles of a person-centred curriculum (27). The framework accommodates pragmatism from both an organisational and learner perspective. From an organisational perspective, not all learning environments have equal resources and so curriculum design may vary between settings even though they aim to incorporate the same components. For example, one setting may offer a six month PCLP within a national academic degree, whilst another setting may be restricted to a five day (non)residential school for leaders of a local organisation. From the learner perspective, the learning and development should be grounded in the realities of their everyday practice, using stories and experiences from practice as well as applying new knowledge in practice. Through the transformative principle, learners should be challenged to critically reflect on the values and assumptions underlying their own being, as well as those embedded within the local environment, before, during and after they experiment with (new) leadership behaviours. The PCLP facilitators should not prescribe learning, but co-construct learning structures and activities so as to accommodate individual as well as collective learning needs and capacities. This focus on learning relationships is guided by the relationism principle of a person-centred curriculum as learners and facilitators learn with and from each other (27).

### 4.2 Purpose and value of the PCLCF in international health care

This is the first Delphi study wherein an international panel of health and social care experts in the field of person-centred leadership, education, practice and research have sought and achieved consensus on the components parts of a person-centred leadership curriculum framework.

The framework brings together and complements existent literature on person-centred leadership development (7–9, 11, 13, 14, 18). It can now support the delivery of leadership programmes designed to enable health care leaders cultivate person-centred cultures within health and social care settings. We would emphasis the importance of paying attention to all four themes during any programme and two core principles: continuous self-awareness and continuous development, as leadership development should be a never-ending-story. As leaders consider how different theories of leadership can be enacted in the messy worlds of practice, where context is dynamic and often less than ideal, they should start to realize that the leadership relationships they build and sustain are in a constant state of being and becoming as they focus on enabling the “coming into their own” of others and self (21). Being and becoming a person-centred leader has no beginning nor end. As responsive and embedded persons, leader values and assumptions (and consequently behaviour) are more likely than not to evolve across time. Therefore the skills and methods acquired whilst engaging in a PCLP based on adult and transformative learning theory, should equip leaders in remaining self-aware and self-determinant as their leadership continues to evolve. When leaders experience the benefits of a supportive, critical yet non-judgmental learning environment based on person-centred learning theory within a PCLP, they should also start to realise the inherent capacity each person has to direct their continued leadership development, and appreciate the mutual benefits of learning with and from others. Additionally, observing how person-centred facilitators on such programmes supports their individual and group learning could also offer learners insight into the skills and methods that they could adopt into their leadership whilst aiming to facilitate the development of person-centredness within the care environments they are leading.

As more leadership development programmes are based on the framework, larger scale, international impact evaluation studies can be designed and conducted to evidence the role of leadership development in cultivating person-centred practice.

### 4.3 Critique of delphi study

Strong consensus was achieved after two rounds commensurate with that report in most Delphi studies in healthcare research (28). In round 1 only 1 component had a consensus score of <80%, and in round 2 only three components were <90%. The speed of consensus forming could be attributed to the decision of the research team to develop a tentative curriculum framework (based on literature study and professional experience and expertise) first, rather than start by seeking participant opinions of which component parts they felt should be included in the curriculum framework. Gathering expert opinions on what should be included first is justified when the research team is small (one to two researchers) or seeking consensus on a relatively new or poorly defined phenomenon. However, our research team was substantial in size (*n* = 7) and existent literature alongside experiential knowledge enabled the co-construction of a tentative curriculum framework with a strong foundation. The fact that the seven members of the research team sought and achieved consensus among themselves on the content of the tentative curriculum and were actively involved in data-analysis of both survey rounds, it could be argued that the final PCLCF is based on consensus among 32 experts (7 researchers +25 participants). Manyara et al. (29) recommend a sample size of 20–30 participants for homogenous stakeholder groups.

Quasi-anonymity, electronic surveying and controlled feedback on consensus between rounds were strategies employed to enhance authentic responses. Participant response (84%) and level of consensus (mode for all components was “strongly agree”) were both high in round 2. Although we sought expertise by the use of participant selection criteria for PCP-ICoP members, it could be argued that the self-selection process is prone to bias and a more heterogenous stakeholder group is desirable. However, recruitment was not restricted to PCP-ICoP members and participants came from a range of countries across the globe and a range of academic, education and practice settings. Representation from Eastern countries and the Americas was lacking. Also, (front-line) clinical leaders who had completed a PCLP and/or can demonstrate lived experience of practicing person-centred leadership, was not identified among panel experts. However, relevance of the curriculum framework for contemporary clinical practice can be assumed as we consider contemporary (research) literature on relational and person-centred leadership.

### 4.4 The position of the PCLCF within contemporary health care leadership literature

The PCLCF pays significant attention to the association between leadership and person-centred cultures. Components 8 and 13 ensure leaders proactively and continually work on developing positive psychosocial environments where relational connectedness grows. It is through this leaders can safely critique their practice against shared visions and standards to enhance person-centred care (components 12, 14 and 17). This aligns with the work of Backman et al. (30), where, in their longitudinal study of Swedish nursing home managers, they confirmed previous study findings showing that leadership is positively related to person-centred care and the psychosocial climate. A possible explanation for this relationship is that person-centred care is a multifaceted, holistic care model requiring whole-system approaches for sustainable change i.e., continuous facilitation, evaluation and synchronised care activities led by local leaders who are sensitive to organisational, professional and workplace cultures and needs (components 14 and 15). Components 8, 11 and 15 in our curriculum relate closely to Backman et al's (30) other findings which suggest that with continued/increased relational connectedness, leaders are able to cultivate a psychosocial climate (components 15) which benefits resident wellbeing and quality of care.

The content of our suggested curriculum alongside the pedagogical principles ensure that leaders are engaged in reflexivity, continuously considering ethical values and person-centred practices (components 1,2, 3, 11, 15 and 18). We see this as a critical prerequisite to leading in person-centred ways and facilitating person-centred care cultures. Backman et al. (30) found that leaders who had only engaged in general leadership education and development programmes did not have as strong an association with person-centred care and psychosocial climate as those who had engaged in leadership development programmes that focused on ethical values and (person-centred and evidence-based) practices alongside systematic thinking and development work. Cardiff et al. (11) similarly found that generating and enacting a shared vision of good care (components 12) is characteristic of effective workplace cultures.

Local leadership is also known to be a significant factor contributing to healthcare staff intention to stay or leave their position, organisation and their profession (9). Our proposed PCLCF includes a number of components (8, 11, 13, 16 and 18) focusing on developing those characteristics known to recruit and retain health and social care staff. For instance, nurses are known to prefer working in settings where they have feel supported by colleagues (components 8) and have the ability to collectively influence their practice (environment) (components 11) (12). A number of components closely align to Cardiff et al's (11) four guiding lights to support front-line healthcare leaders: enabling professional practice autonomy of staff (components 11,13,16 and 18); supporting staff professional growth and development (components 9, 11 and 16); cultivating healthful workplace cultures (components 14- 18) and fostering relational connectedness with and among staff (components 8, 11 and 16). They also found that the mutuality and reciprocity emergent through relational leadership also enabled leader wellbeing and growth (components 4–8). In their scoping review of the impact of relational leadership, Tuepker et al. (31) found that relational leadership positively impacts staff burnout, engagement, well-being, workplace trauma, workplace climate and communication, as well as outpatient costs, quality care measures, patient satisfaction and outcomes. Based on six different intervention studies, they also conclude that relational leadership can be taught and cultivated within healthcare organisations. However, like us, they found no clearly reported curriculum framework for relational leadership either. All of their included studies did use small group learning with time to reflect and/or problem solve collectively, as well as workshops on relevant topics such as team dynamics and conflict transformation. This mix is reflected in the pedagogical principles of the PCLCF (components 19–24). 2.3.5 Delivery of the PCLCF considerations.

In terms of programme duration we recommend that this PCLCF should not be prescriptive and so accommodate contextualisation of the programme. For instance, some programmes may be longer/shorter than others due to resource availability, or include different themed workshops based on learner needs and interests. Based on their findings that all six interventions took place across several months or longer, Tuepker et al. (31) do suggest that leadership development requires duration in order for embodiment and practice transformation to take place. Anker-Hansen et al's (32) review also concludes that leaders need to embody person-centered values (components 1–3) and role model them as they exercise influence vertically and horizontally (component 10) to foster sustained person-centred practice. However, we would argue than growth and development can continue outside formal educational programmes if learners are equipped with the skills and tools to self-direct their learning and development. Hence the PCLCF principles of offering choice (component 20), using multiple sources of knowledge and pedagogical methods (component 21), using high challenge and high support (component 22) and addressing the real world of practice (component 24) are critical to success.

Our collective knowledge as experienced facilitators of leadership development has also taught us that although transformational, learners unused to more self-directed and co-constructed learning, may find it disorientating initially and struggle to move away from more prescriptive forms of learning. This is why in the PCLCF has component 22. The relational nature of person-centred facilitation gives space for issues to be addressed, creates time for learners to grow skills in adult learning and to “let go” of what came before to make space (emotionally and psychologically) for honesty, braveness and courage in identifying what needs to evolve. Preferably, a PCLP would be paced across several months at least and ideally 12 months with monthly contact. Cardiff & Frost's (8) evaluation a 12 month leader development programme illustrates how monthly contact using a co-operative inquiry structure and process can transform participant thinking and doing.

### 4.5 Potential impact of future person-centred leaders

The PCLCF draws on the assumption that leadership is a suite of relational practices between persons (21). Engaging in such leadership practices enables a coming into own for those being led as well as the leader themselves, regardless of the job descriptions. Cardiff's (33) description of PCL in practice also describes in more detail how the leader role can move fluidly between a leader and associate when they have embodied person-centredness within a person-centred culture. We have therefore ensured through the inclusion of component 9, leaders can consciously, and in the moment, consider their position in relation to associates and as such continually strengthen trust and empowerment. Our careful attention to relational practices in the curriculum aligns with the definitions and literature on relational leadership theory which reconceptualise leadership from a hierarchical relationship between “leaders” and “followers” to a set of “relational practices” between persons. In doing so, relational leadership recognises leadership practices at every level within an organisation and so releases the potential of all employees to influence situations within their field of expertise and contribute to an ethical climate (34). This insight raises the question what impact a leadership development programme, based on the PCL curriculum framework, would have on healthcare practitioner perceived autonomy and agency in fostering person-centred cultures and care. Indeed, Cardiff et al's (11) study on workplace cultures states that focusing on the development of collective leadership (a dynamic, emergent and interactive influence process among team members who selectively utilize one another's skills and expertise to direct, motivate and support whole team performance) is one recommendation for cultivating effective workplace cultures where healthcare workers want to work (component 11). Jönsson et al. (35) also found that nurses in residential care felt that leading person-centred care entails continuously striving to live and be person-centred in both care and collegial relationships (components 1- 8), as well as taking ownership and sense-making person-centredness in a complex care environment (components 14–18).

## 5 Conclusion

This study starts to address the gap in knowledge if health care leaders are to support the delivery of person-centred health and social care more effectively. The evidence is clear that healthcare leaders have a vital role to play in cultivating person-centred cultures and care, where all feel respected, valued and can flourish. Whilst hierarchically placed leaders, such as (ward/department/executive) managers, may focus primarily on cultivating person-centred practices, clinical leaders such as nurses and allied health practitioners may focus on person-centred care. Collaboratively and collectively, managers and clinical leaders should be focusing on developing person-centred cultures of care through role modelling person-centredness and actively facilitating it's co-creation. How health care leaders can be supported in becoming more person-centred and cultivating such cultures is less well understood. Whilst numerous leadership models are being employed and leadership development programmes being delivered, there is no international curriculum framework for person-centred leadership development, and so robust evaluation cannot take place.

The PCLCF framework presented in this paper has a strong theoretical foundation and consensus on the content was achieved through a robust Delphi study using an international panel of experts within healthcare education, research and healthcare administration. The framework offers curriculum developers and educators 18 components to be covered in the programme as well as six guiding principles on how to deliver the programme. The absence of contextual details such as length and structure of the programme, makes it possible to deliver a balanced person-centred leadership development programme that fits local context and learner needs and capacity. It is in itself person-centred. Whilst the curriculum framework has been robustly developed, continued rounds of consensus seeking and critical dialogue on its applicability across various healthcare contexts across the globe, would fit within the constructivist epistemology underpinning the Delphi method.

The health care context in which leaders work is increasingly demanding, complex and in a constant state of flux. The PCLCF seeks to pay attention to this by supporting leaders to consider their positionality and influence within the workplace and boost their self-efficacy to lead with moral intent. Only by doing so can leaders effectively balance their efforts to create the conditions necessary for person-centred care delivery, stay consistent and authentic in their leadership approach and pay attention to their own and others’ wellbeing.

## Data Availability

The original contributions presented in the study are included in the article/Supplementary Material, further inquiries can be directed to the corresponding author/s.
